# Relationship Between Coronavirus-Related eHealth Literacy and COVID-19 Knowledge, Attitudes, and Practices among US Adults: Web-Based Survey Study

**DOI:** 10.2196/25042

**Published:** 2021-03-29

**Authors:** Lawrence An, Elizabeth Bacon, Sarah Hawley, Penny Yang, Daniel Russell, Scott Huffman, Ken Resnicow

**Affiliations:** 1 Center for Health Communications Research Rogel Cancer Center University of Michigan Ann Arbor, MI United States; 2 Division of General Medicine School of Medicine University of Michigan Ann Arbor, MI United States; 3 VA Center for Clinical Management Research VA Ann Arbor Healthcare System Ann Arbor, MI United States; 4 Google Mountain View, CA United States; 5 Department of Health Behavior & Health Education University of Michigan School of Public Health Ann Arbor, MI United States

**Keywords:** internet, digital health, eHealth, eHealth literacy, coronavirus, COVID-19, knowledge, conspiracy beliefs, protective behaviors, social distancing, survey, health communication, attitude, behavior

## Abstract

**Background:**

During a global pandemic, it is critical that the public is able to rapidly acquire new and accurate health information. The internet is a major source of health information. eHealth literacy is the ability of individuals to find, assess, and use health information available on the internet.

**Objective:**

The goals of this study were to assess coronavirus-related eHealth literacy and examine the relationship between eHealth literacy and COVID-19−related knowledge, attitudes, and practices (KAPs).

**Methods:**

We conducted a web-based survey of a representative sample of 1074 US adults. We adapted the 8-item eHealth Literacy Scale to develop the Coronavirus-Related eHealth Literacy Scale (CoV-eHEALS) to measure COVID-19−related knowledge, conspiracy beliefs, and adherence to protective behaviors (eg, wearing facial masks and social distancing). Our analyses identified sociodemographic associations with the participants’ CoV-eHEALS scores and an association between the CoV-eHEALS measure and COVID-19 KAPs.

**Results:**

The internal consistency of the adapted CoV-eHEALS measure was high (Cronbach α=.92). The mean score for the CoV-eHEALS was 29.0 (SD 6.1). A total of 29% (306/1074) of the survey participants were classified as having low coronavirus-related eHealth literacy (CoV-eHEALS score <26). Independent associations were found between CoV-eHEALS scores and ethnicity (standardized β=–.083, *P*=.016 for Black participants) and education level (standardized β=–.151, *P*=.001 for participants with high-school education or lower). Controlling for demographic characteristics, CoV-eHEALS scores demonstrated positive independent associations with knowledge (standardized β=.168, *P*<.001) and adherence to protective behaviors (standardized β=.241, *P*<.001) and a negative association with conspiracy beliefs (standardized β=–.082, *P*=.009).

**Conclusions:**

This study provides an estimate of coronavirus-related eHealth literacy among US adults. Our findings suggest that a substantial proportion of US adults have low coronavirus-related eHealth literacy and are thus at a greater risk of lower and less-protective COVID-19 KAPs. These findings highlight the need to assess and address eHealth literacy as part of COVID-19 control efforts. Potential strategies include improving the quality of health information about COVID-19 available on the internet, assisting or simplifying web-based search for information about COVID-19, and training to improve general or coronavirus-specific search skills.

## Introduction

During a global pandemic, it is critical that members of the public are able to rapidly acquire new and accurate health information [1-4]. This includes information about the causative agent, transmission and course of the disease, as well as prevention and treatment [5,6]. It is also important to be able to avoid misinformation that might discourage protective behaviors or even encourage actions that lead to self-harm. This includes specific mistaken beliefs (eg, the new disease is no more serious than common existing infections) and also broader conspiracy theories (ie, unsubstantiated and implausible assertions that hidden forces in control of the society have created or are using the pandemic to extend their authority) that can interfere with or undermine individual or public responses to the pandemic. Research continues to identify deficits in the public’s knowledge of key facts regarding the current COVID-19 pandemic, particularly among underserved groups that bear a disproportionate burden in terms of the number of COVID-19 cases and deaths [7-10]. The prevalence and negative impact of misinformation and the spread of conspiracy beliefs during the current pandemic (eg, spread of the “Plandemic” viral video) have also been increasingly recognized [6,11-14]. 

The internet has become a major source of health information for the public [15-19]. eHealth literacy refers to the ability of individuals to find, assess, and effectively use health information available on the internet [20]. The eHealth Literacy Scale (eHEALS) is one of the most commonly used measures to assess eHealth literacy and has been shown to be reliable and valid across a range of health conditions and populations [21-32]. eHealth literacy may be particularly important during the current pandemic because some widespread disease-control strategies (eg, social distancing) can limit in-person contact and reduce transmission of key information through these channels. Several authors have highlighted the importance of considering eHealth literacy in the response to the current global COVID-19 pandemic [1-4]. A prior study has demonstrated an association between higher receipt of information about COVID-19 available on the internet and increased engagement in personal protective behavior against COVID-19, such as handwashing, wearing facial masks, and avoiding social gatherings [33].

The goals of this study were to: (1) understand the ability of individuals to identify, assess, and effectively utilize health information about the coronavirus available on the internet and (2) determine how this ability might be associated with COVID-19–related knowledge, attitudes, and practices (KAPs). We performed a web-based survey of a nationally representative sample of US adults to achieve these goals.

## Methods

### Measures

For each of the following measures, specific survey items were presented to the survey respondents in random order. 

#### Coronavirus-Related eHealth Literacy

We slightly modified items from the well-established eHEALS [20] to focus specifically on health information available on the internet *about the coronavirus*. The resulting 8-item measure assesses an individual’s self-rated ability (answered on a 5-point Likert scale) to use the internet to find and utilize health information about the coronavirus. The specific items of this coronavirus-related eHEALS (CoV-eHEALS) were as follows:

1. I know what health resources about coronavirus are available on the internet. 
2. I know where to find helpful health resources about coronavirus on the internet.
3. I know how to find helpful health resources about coronavirus on the internet.
4. I know how to use the internet to answer my questions about my health and coronavirus.
5. I know how to use the health information about coronavirus I find on the internet to help me.
6. I have the skills I need to evaluate the health resources about coronavirus I find on the internet.
7. I can tell high-quality health resources from low-quality health resources about coronavirus on the internet.
8. I feel confident in using information about coronavirus from the internet to make health decisions.

An overall CoV-eHEALS score was computed based on the sum of the scores for each item (range 8-40). The internal consistency of the CoV-eHEALS measure was 0.92, and this was not improved by the deletion of any specific item. Some prior studies have also defined cut-off points to characterize respondents as having low versus high eHealth literacy [22,27,29]. Consistent with this work, we categorized respondents, based on their total CoV-eHEALS score, as having low (score <26) or high (score ≥26) coronavirus-related eHealth literacy.

#### COVID-19 KAPs

We assessed the survey respondents’ COVID-19 KAPs by using the following measures. 

##### Knowledge

We created a 7-item scale based on common key facts related to COVID-19, recognized as of May 2019 [5,6]. Each item was answered on a 5-point scale ranging from “Definitely false” to “Definitely true.” The specific items of this scale were as follows:

1. Coronavirus can be easily spread from one person to another.
2. Many thousands of people have died from coronavirus.
3. A vaccine is not yet available for the coronavirus.
4. Most people already have immunity to coronavirus.
5. Symptoms of coronavirus are always visible.
6. There are effective treatments for coronavirus that can cure most people.
7. Having coronavirus is about as dangerous as having the flu.

After reverse-coding of items 4-7, we created an overall knowledge score based on a mean of the scores for each item (range 1-5). The internal consistency of this knowledge measure was 0.78, and this was not improved by the deletion of any specific item.

##### Conspiracy Beliefs

We developed a brief, 3-item scale based on prior studies on COVID-19 and other health issues [14,34]. The scale is intended to measure conspiracy beliefs regarding the coronavirus rather than a generalized conspiracy trait or worldview. Each item was answered along a 5-point continuum ranging from “Definitely false” to “Definitely true.” The specific items of this scale were as follows:

1. The real truth about coronavirus is being kept from the public.
2. People in power are using coronavirus as an excuse to monitor and control the public.
3. The media is making coronavirus seem more dangerous that it really is. 

We computed a mean of the response to these 3 items to create a conspiracy score (range 1-5). The internal consistency of this conspiracy measure was 0.74, and this was not improved by the deletion of any specific item.

##### Protective Behavior Adherence Score

We examined the frequency of 7 self-reported behaviors practiced by the survey respondents over the past week, all of which are recommended for reducing the risk of transmitting and/or acquiring COVID-19 [35]. Each item was answered on a 5-point continuum: “Rarely or never,” “Some of the time,” “Most of the time,” “Almost all of the time,” and “All of the time.” Our measure shares many topics in common to a recently described measure of COVID-19 infection prevention behaviors [36]. The specific items of this scale were as follows: 

1. Avoiding touching my face.
2. Keeping my hands clean (eg, washing longer with soap and water, using hand sanitizer).
3. Keeping things clean in my home (eg, phone, refrigerator, doorknobs).
4. Staying home as much as possible.
5. Wearing a mask or face covering when I go out of the house.
6. Staying at least six feet (about 3 steps) away from people I don’t live with.
7. Avoiding gatherings or groups of other people.

We computed a mean of the response to these items to create a protective behavior adherence score (range 1-5). The internal consistency of this positive protective behaviors index was 0.85, and this was not improved by the deletion of any specific item.

### Demographic Characteristics

Information on the demographics of the survey respondents, including age, gender, race or ethnicity, level of education, income, and political party affiliation, was obtained. Gender was initially assessed using 5 categories: male, female, transgender (identify as male), transgender (identify as female), and other. The responses were then collapsed into 2 categories (“identify as male” and “identify as female”). Race or ethnicity was coded as White, Black, Hispanic, multiracial, and other (which included American Indian, Asian, and other). Education was initially assessed with 10 strata, which were collapsed into 4 categories: none through high school or general education diploma, postsecondary (eg, trade school, some college, or associates), bachelor’s, and advanced degree (eg, masters, doctoral or professional). Income was assessed with 9 strata, ranging from less than US $20,000 to more than US $150,000.

### Survey Administration

The full survey assessed a range of individual and household characteristics, attitudes, and behaviors related to the COVID-19 pandemic. Surveys were completed through the Qualtrics web-based platform using a sample provided by Dynata [37]. Dynata’s research panel comprises an opt-in list of over 60 million individuals globally. For this study we requested a nationally representative sample of 1000 US adults aged 18 years and above. Quotas were used to approximate national rates for age, gender, race, income, and US region. The survey was conducted as open enrollment, whereby eligible panel members who log into the Dynata website were offered a chance to participate in this survey. Participants received modest compensation (approximately US $1) from Dynata for completing the survey.

 During the last week of May 2020, a total of 2272 individuals clicked on our survey invitation link, of which 187 did not complete an age screener item or consent, and 609 were ineligible for the survey or refused consent. This yielded 1476 complete survey responses from age-eligible, consenting individuals. To ensure the quality of the respondent data, we further excluded 402 survey responses based on either of two criteria. First, we excluded 375 survey responses from individuals who completed the entire survey in less than 10 minutes (the minimum time we considered needed to complete a valid survey). The mean time for survey completion for these excluded respondents was 5.4 (SD 3.3) minutes. Second, we excluded 27 survey responses from individuals who answered all items within a 16-item block of items assessing attitudes (and perceived norms) toward the pandemic with an identical response. This is the equivalent of clicking down an entire column (eg, all “Strongly Agree” or “Disagree” responses) for all items. Because some of the 16 items in this section were worded in the positive direction (eg, *Social distancing has slowed the spread of the coronavirus*) and the others, in the negative direction (eg, *Social distancing is not really doing much good*), we considered these “response set” patterns contradictory and a sign of poor-quality survey responses. Thus, we finally considered 1074 surveys for the present analyses. The mean time to complete the survey was 25.3 (range 10.1-117.1) minutes for the included participants.

### Hypotheses

We have two sets of hypotheses regarding the relationship between CoV-eHEALS scores and the participants’ demographic characteristics and COVID-19–related KAPs.

#### Hypothesis 1

We expect to find significant associations between CoV-eHEALS scores and demographic characteristics. Specifically, we have the following expectations:

- Hypothesis 1a: CoV-eHEALS score will be negatively associated with age (ie, it will be lower among older individuals). This is based on several prior studies that found lower general eHEALS scores among older individuals [26,27,29,30].
- Hypothesis 1b: CoV-eHEALS score will be lower among ethnic minority groups. This is based on prior studies that have reported lower engagement with health information available on the internet or lower general eHEALS scores among minority populations [17,19,27,30].
- Hypothesis 1c: CoV-eHEALS score will be positively associated with educational attainment (ie, it will be higher among those who report completing higher formal education). This is based on several prior studies that identified this relationship between educational attainment and the general eHEALS measure [23,26,27,29,30].

#### Hypothesis 2

We expect to find significant associations between CoV-eHEALS scores and COVID-19 KAPs. Specifically, we have the following expectations:

- Hypothesis 2a: CoV-eHEALS score will be positively associated with COVID-19–related knowledge. This is based on prior studies showing a positive association between the general eHEALS measure and disease-specific knowledge or perceived understanding and knowledge of personal health issues [29,31].
- Hypothesis 2b: CoV-eHEALS score will be negatively associated with conspiracy beliefs. Mistrust of traditional information sources (eg, government, public health agencies, and mainstream media) is a core characteristic of individuals who hold conspiracy beliefs. We believe there is likely a negative association between a person’s trust in these information sources and their confidence that they can find, assess, and use health information available on the internet.
- Hypothesis 2c: CoV-eHEALS score will be positively associated with adherence to behaviors that protect from COVID-19. This is based on prior studies showing more positive health behaviors (eg, healthy lifestyle and engagement in cancer screening) among individuals with higher general eHEALS scores [32,38].

### Statistical Procedures

For hypothesis 1, we examined the relationship between demographic variables and the CoV-eHEALS score. Age and income (represented as 9 income strata) were examined as continuous variables. Gender, ethnicity, and educational attainment were examined as categorical variables. We first examined these associations separately using Pearson’s correlation to examine the association between CoV-eHEALS score and continuous variables (eg, age and income) and analysis of variance to examine the association between CoV-eHEALS score and categorical variables (eg, gender, ethnicity, and educational attainment). We then examined the independent association between demographic variables and CoV-eHEALS score by using a linear regression model with CoV-eHEALS score as the dependent variable and demographic characteristics as the independent variables (with dummy coding of gender, ethnicity, and education).

For hypothesis 2, we examined the association between CoV-eHEALS score and COVID-19 knowledge, conspiracy beliefs, and protective behaviors. We performed multivariate analysis of variance (MANOVA) to simultaneously assess the relationship between these three dependent variables (scores for knowledge, conspiracy belief, and protective behavior adherence) and our main variable of interest (ie, low vs high CoV-eHEALS scores), while controlling for demographic characteristics as covariates (with age and income as continuous variables and dummy coding for gender, ethnicity, and education). To further illustrate the relationship between CoV-eHEALS scores and COVID-19 KAPs, we created simplified composite variables to represent each KAP measures. For knowledge, we computed a sum of the total number of knowledge items answered correctly (ie, answered “Definitely true” or “Probably true” for knowledge items 1-3 and “Definitely false” or “Probably false” for knowledge items 4-7) by each respondent (range 0-7). For conspiracy beliefs, we computed a sum of the total number of conspiracy items rejected (ie, answered “Definitely false” or “Probably false”) by each respondent (range 0-3). For protective behaviors, we computed a sum of the total number of behaviors for which the respondent reported routine engagement (eg, answered “Always” or “Almost Always”). We then compared the distribution of these compositive variables for respondents classified as having low versus high CoV-eHEALS scores by using chi-square tests to assess statistical significance.

All analyses for this study were performed using SPSS software (version 25; IBM Corp).

### Ethical Review

This survey study was reviewed and judged to be exempt (survey without identifying information) by the University of Michigan’s institutional review board. 

## Results

The demographic characteristics of the study participants and their CoV-eHEALS and COVID-19 KAP scores are shown in Table 1. The sample comprised 55.6% (575/1074) female, 69.9% (723/1074) White, 8.1% (84/1074) Black, 9.2% (95/1074) Hispanic, and 6.3% (65/1074) multiracial participants. Their mean age was 47.3 (SD 17.1) years. Their mean CoV-eHEALS score was 29.0 (SD 6.1), and their mean scores for the COVID-19 KAP measures were as follows: knowledge 3.8 (SD 0.8), conspiracy beliefs 2.9 (SD 1.1), and protective behaviors index 3.9 (SD 0.9).

Results of the analyses related to hypothesis 1 (ie, Associations between CoV-eHEALS score and demographic characteristics) are shown in Tables 2 and 3. Using bivariate comparison, we found a significant association between the CoV-eHEALS score and income and educational attainment (Table 2). The correlation between CoV-eHEALS score and income was positive (*r*=0.087, *P*=.005) indicating individuals with higher income have higher coronavirus-related eHealth literacy. The direction of the relationship between CoV-eHEALS score and education was similar—individuals with higher educational attainment reported higher CoV-eHEALS scores. Results from a multivariate liner regression model are shown in Table 3. In this model, there are independent associations between CoV-eHEALS scores and ethnicity and educational attainment. The CoV-eHEALS score was lower for Black participants (standardized β=–.083, *P*=.02) than for White participants (the reference group). The CoV-eHEALS score was lower among participants who completed education only up to a high-school degree (standardized β=–.151, *P=*.001) than among those with advanced degrees (the reference group). In the regression model, the association with income was no longer significant. The association between CoV-eHEALS score and age was not significant in either the bivariate (*r*=0.009, *P*=.79) or multivariate (standardized β=–.038, *P=*.29) analysis.

**Table 1:** Characteristics of study participants (N=1074) and their mean scores for various study measures.

Variable			Value, n (%)
**Age (years), n (%)**			
	18-35	304 (29.5)	
	36-50	263 (25.6)	
	51-65	277 (26.9)	
	≥65	185 (18)	
**Income (US $)** **, n (%)**			
	<30,000	291 (28.1)	
	30,000-74,999	397 (38.4)	
	≥75,000	346 (33.5)	
**Gender** **, n (%)**			
	Male	459 (44.4)	
	Female	575 (55.6)	
**Race or ethnicity, n (%)**			
	White	723 (69.9)	
	Black	84 (8.1)	
	Hispanic	95 (9.2)	
	Multiracial	65 (6.3)	
	Other	67 (6.5)	
**Education, n (%)**			
	Up to high school or GED^a^	225 (21.8)	
	Postsecondary (eg, trade school, some college, or associates)	326 (31.6)	
	Bachelor’s degree	310 (30)	
	Advanced degree (eg, Masters, Doctoral or Professional)	172 (16.7)	
**Scores, mean (SD)**			
	Coronavirus-related eHealth Literacy Scale (range 8-40)	29.0 (6.1)	
	Knowledge (range 1-5)	3.8 (0.8)	
	Conspiracy beliefs (range 1-5)	2.9 (1.1)	
	Positive behavior adherence (range 1-5)	3.9 (0.9)	

^a^GED: Tests of General Educational Development.

**Table 2:** Bivariate association between demographic characteristics and coronavirus-related eHealth literacy.

Variable			eHealth literacy score, mean (SD)	*P* value
**Gender**				.47
	Male	29.2 (6.3)		
	Female	28.9 (5.9)		
**Ethnicity**				.21
	White	29.1 (6.0)		
	Black	27.6 (5.7)		
	Multiracial	28.9 (6.3)		
	Hispanic	29.4 (5.9)		
	Other	29.3 (7.1)		
**Education**				<.001
	Up to high school or GED^a^	27.6 (6.6)		
	Postsecondary (eg, trade school, some college, or associates)	28.8 (6.0)		
	Bachelor’s degree	29.9 (5.7)		
	Advanced degree (eg, Masters, Doctoral or Professional)	30.0 (5.5)		

^a^GED: Tests of General Educational Development.

**Table 3:** Independent association between demographic characteristics and coronavirus-related eHealth literacy.

Variable		Standardized β coefficient	*P* value
Age (continuous)		–.038	.29
Income (continuous, 9 strata)		.023	.21
**Gender**			
	Male	Ref^a^	
	Female	–.003	.92
**Ethnicity**			
	White	Ref	
	Black	–.083	*.02* ^b^
	Multiracial	–.018	.60
	Hispanic	–.006	.86
	Other	–.014	.68
**Education**			
	Up to high school or GED^c^	–.151	*.001* ^b^
	Postsecondary (eg, trade school, some college, or associates)	–.079	.09
	Bachelor’s	–.007	.87
	Advanced degree (eg, Masters, Doctoral or Professional)	Ref	

^a^Ref: reference value.

^b^Italicized values indicate statistical significance.

^c^GED: Tests of General Educational Development.

Results of the analyses related to hypothesis 2 (ie, association between CoV-eHEALS and COVID-19 KAP scores) are shown in Table 4. Among our respondents, 29% (306/1074) were classified as having low coronavirus-related eHealth literacy (ie, total CoV-eHEALS score <26). Results from the MANOVA model show a significant association between CoV-eHEALS score and all three (COVID-19 knowledge, conspiracy beliefs, and adherence to protective behaviors) dependent variables (*F*_3,1013_=20.89, *P*<.001; Wilks Λ=0.94, partial η^2^=0.058). When adjusting for sociodemographic differences, respondents with higher (vs lower) CoV-eHEALS scores had higher mean scores for knowledge and protective behaviors adherence and a lower mean score for conspiracy beliefs.

**Figure 1:**
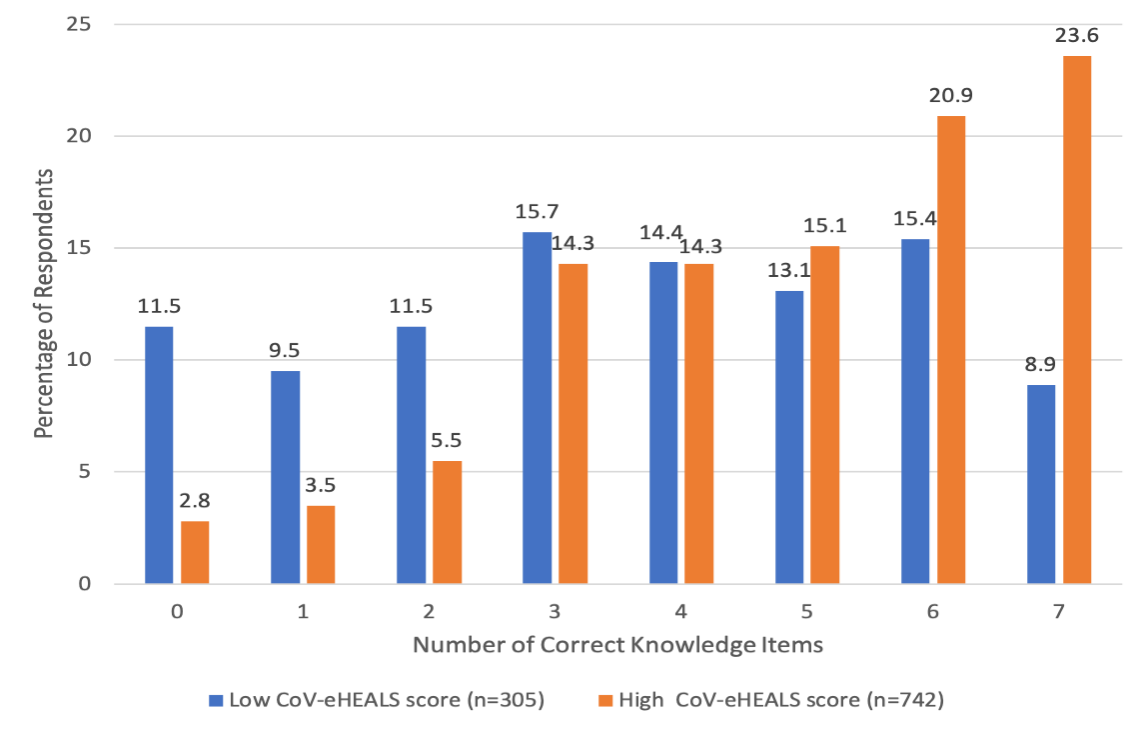
Number of correct knowledge items by coronavirus-related eHealth literacy.

**Figure 2:**
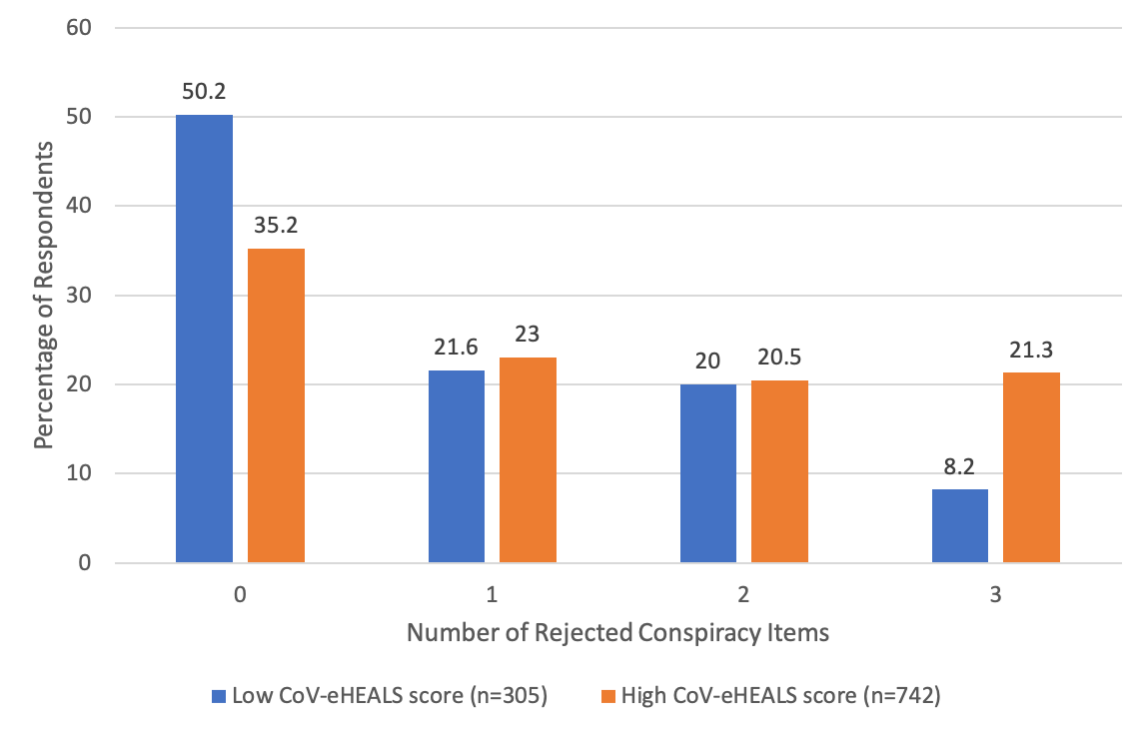
Number of rejected conspiracy items by coronavirus-related eHealth literacy.

**Figure 3:**
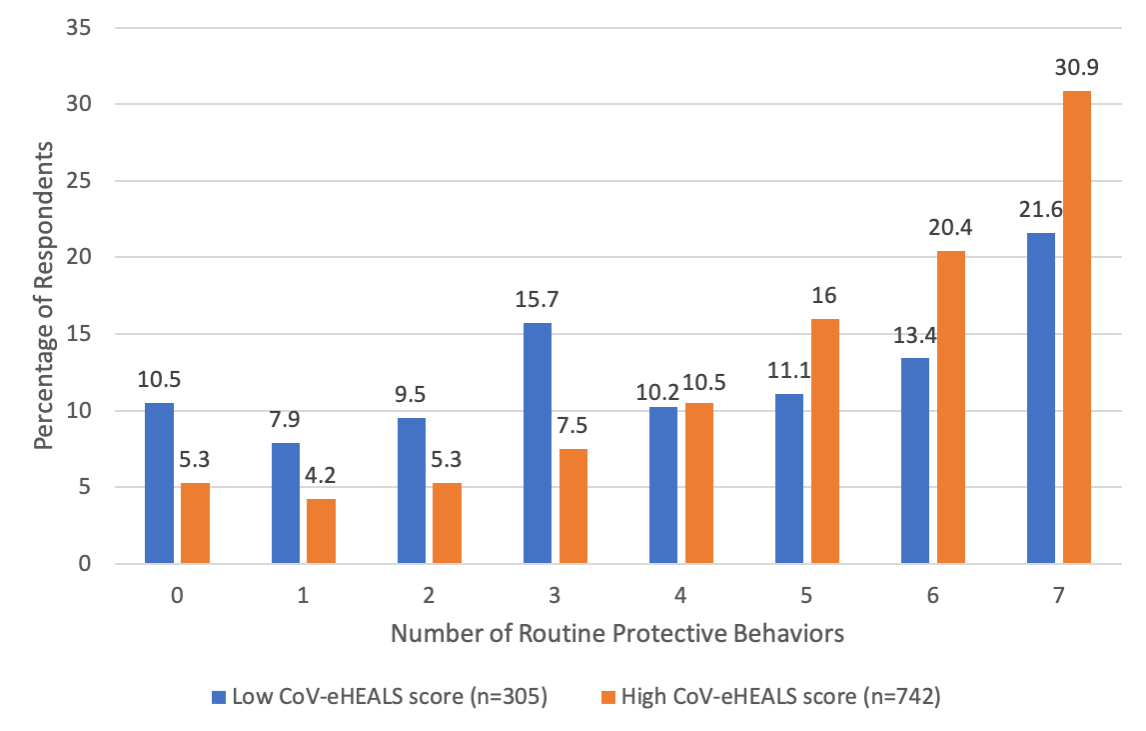
Number of routine protective behaviors by coronavirus-related eHealth literacy.

The nature of the relationship between CoV-eHEALS and COVID-19 KAP scores is further illustrated in Figures 1-3. Figure 1 compares the number of knowledge items answered correctly by respondents with low versus high CoV-eHEALS scores. For those with low CoV-eHEALS scores, nearly 33% (99/305) correctly answered 2 or fewer knowledge items, and less than 10% (27/305) correctly answered all 7 items. For those with high CoV-eHEALS scores, less than 20% (88/742) correctly answered 2 or fewer knowledge items and nearly 25% (175/742) correctly answered all 7 items. The difference in the distribution of the number of correct answers on these 7 knowledge items was statistically significant (*P*<.001).

Figure 2 compares the number of rejected conspiracy items (ie, participants who assessed the conspiracy item as “Definitely False” or “Probably False”) for participants with low versus high CoV-eHEALS scores. For those with low CoV-eHEALS scores, approximately 50% (153/305) did not reject any of these items, whereas only 8% (25/305) of them rejected all 3 items. For those with high CoV-eHEALS scores, 35% (261/742) did not reject any of these items, whereas 21% (158/742) rejected all 3 items. The difference in the distribution of the number of rejected conspiracy items was statistically significant (*P*<.001).

Figure 3 compares the number of protective behaviors routinely reported by the participants (ie, “Always” or “Almost Always” engaging in this practice) with low versus high CoV-eHEALS scores. For those with low CoV-eHEALS scores, nearly 30% (85/305) reported engaging in 2 or fewer behaviors, and a similar proportion of the participants (107/305, 35%) reported routine practice of 6 or all 7 of the protective behaviors. For those with high CoV-eHEALS scores, about 15% (109/742) of the participants reported engaging in 2 or fewer behaviors, whereas just over 50% (487/742) reported routine practice of 6 or all 7 of the protective behaviors. The difference in the distribution of the number of routine protective behaviors was statistically significant (*P*<.001).

**Table 4:** COVID-19 knowledge, conspiracy beliefs, and protective behaviors for respondents with low and high coronavirus-related eHealth literacy.

CoV-eHEALS^a^ score	Estimated mean score^b^ (SE)		
	Knowledge	Conspiracy beliefs	Protective behaviors
Low score (n=298)	3.6 (0.040)	3.0 (0.064)	3.6 (0.049)
High score (n=729)	3.9 (0.025)	2.8 (0.040)	4.0 (0.031)
*P* value	<.001	.03	<.001

^a^CoV-eHEALS: coronavirus-related eHealth literacy scale.

^b^Estimated means adjusted for age, income, gender, ethnicity, and education level. Overall multivariate analysis of variance model; Box M=53.35; F=8.86; *P*<.001.

## Discussion

The principal findings of the study show a clear and consistent association between higher coronavirus-related eHealth literacy and greater knowledge, lower conspiracy beliefs, and greater engagement in protective behaviors. The mean CoV-eHEALS score used in this study was similar to those used for the general eHEALS in several population samples [23,26,27]; it was somewhat higher than that reported among some disease-specific groups (eg, cardiovascular disease, lung cancer, patients with HIV) [22,24,39]. It is possible that widespread public attention, media coverage (including content available on the internet), and concerted effort of major public health organizations to disseminate health information on the internet (eg, World Health Organization and the Centers for Disease Control) during the current pandemic contributed to this finding. 

It is important to acknowledge that we administered a modified version of the eHEALS measure that was specific to information about coronavirus (ie, CoV-eHEALS). Although we recognize that this is not typical practice for evaluation of eHealth literacy, we believe this was appropriate given the critical need to assess and understand the ability of individuals and the public to find, assess, and use information available on the internet that is specific to the coronavirus during the current COVID-19 pandemic. The findings we report for this CoV-eHEALS measure are consistent with those recently reported by other teams that administered the general eHEALS measure as part of pandemic-related studies. For instance, a study by Do et al [40] of health care workers in Vietnam reported a significant positive association between participants’ general eHEALS score and their self-reported adherence to occupational infection prevention and control measures. In a national web-based survey of internet users in China, Li and Liu [41] found a significant association between the general eHEALS measure and self-reported practice of protective behaviors against COVID-19 [41]. Future work could examine the relation between CoV-eHEALS and general eHEALS measures and COVID-19−related KAPs.

The results of this study largely support our first set of hypotheses regarding the association between CoV-eHEALS scores and demographic characteristics. Multivariate analyses showed that Black participants had lower CoV-eHEALS scores than White participants (hypothesis 1b). This finding is consistent with some prior studies that have found low general eHEALS scores and a low frequency of seeking health information on the internet among ethnic minority groups [15,17,19,27]. The finding of lower CoV-eHEALS scores among Black participants is of particular concern given the recognized disparities in the impact of COVID-19 on minority groups. Moreover, our finding of lower CoV-eHEALS scores among those with lower educational attainment (hypothesis 1c) is consistent with the same association observed in multiple previous studies using the general eHEALS measure [23,26,27,29,30]. When considered along with the finding of lower CoV-eHEALS scores among Black respondents, this finding reinforces the continued need to address COVID-19−related health disparities that place an undue burden on underserved and disadvantaged groups. Contrary to our original hypothesis 1a, we did not find an association between the participants’ CoV-eHEALS scores and age. This finding is in contrast with a number of prior studies that have identified more advanced age as a predictor of lower general eHEALS scores. The lack of a decrease in CoV-eHEALS score among older participants is somewhat reassuring, particularly given that older adults are at a greater risk of serious illness or death due to COVID-19.

Our study findings also consistently support our second set of hypotheses regarding the association between coronavirus-related eHealth literacy and COVID-19 KAPs. Our analyses showed a significant association in the expected directions between the CoV-eHEALS measure and COVID-19 knowledge, conspiracy beliefs, and engagement in protective behaviors. In considering these findings, it is important to recognize that self-efficacy is a central concept underlying the development of the general eHEALS and, consequently, also for this adapted CoV-eHEALS measure. Although the assessment of self-efficacy is a critical aspect of many major theories of health behavior, it is also recognized that individuals commonly overestimate their abilities to perform more complex tasks [42-45]. For the general eHEALS measure, studies examining the association between self-reported eHealth literacy and the actual performance of functional measures of internet skills have reported variable results. Van Der Vaart and colleagues [28] noted that, in a small sample of patients with rheumatic disease, an individual’s eHEALS score was not consistently related to their actual performance of internet skills. In contrast, Neter and Brainin [46] found a positive, if modest, association between the self-reported general eHEALS measure and observed performance on health-related internet tasks. The nature of the relationship observed between our CoV-eHEALS measure and knowledge provide further insights into this issue. The overall positive and independent association between the CoV-eHEALS and knowledge scores lends support to the validity of the eHEALS approach. At the same time, specific findings also suggest that individuals may overestimate their abilities to some degree. Among those with high CoV-eHEALS scores (ie, high confidence in their own ability to find, assess, and use information about coronavirus on the internet), only about 1 in 4 participants was able to correctly answer all 7 items on our COVID-19 knowledge scale.

The relationship observed between the CoV-eHEALS measure and conspiracy beliefs also warrants further discussion. For our study participants, the mean score on the conspiracy beliefs scale was 2.9, which indicates that, on average, our sample was “unsure” about the truth or falsehood of these statements. Although differences in the wording of questions and format preclude direct comparisons, other studies have reported high rates of endorsement of COVID-19 conspiracy beliefs [47,48]. Our finding of greater rejection of conspiracy beliefs among those with higher CoV-eHEALS scores is consistent with the work of Richtering and colleagues [22] who found a positive association between the eHEALS score and specific facets of a general health literacy measure, including the ability to perform “critical appraisal” of information available on the internet. Although the negative association between CoV-eHEALS and conspiracy scale scores observed in our study is encouraging, it is important to recognize that even among respondents who could be considered as having higher CoV-eHEALS scores (ie, total score ≥26), fewer than half of the participants clearly rejected two or more of the conspiracy items. One study, involving a national survey in Poland, actually reported greater acceptance of conspiracy beliefs among individuals with higher general eHealth literacy [49]. In considering the relationship between CoV-eHEALS scores or general eHEALS scores and these beliefs, it is important to recognize the complex interplay of factors that influence conspiracy thinking such as underlying social orientation (eg, individualist vs collectivist), perception of power or powerlessness, ideology or political affiliation, and media consumption [48,50]. All these factors highlight the importance of considering a broader framework for managing the COVID-19 infodemic. Eysenbach [4] recently presented an “information cake” model that includes building eHealth literacy (and general scientific literacy) along with information monitoring, encouraging knowledge refinement and information quality management, and accurate and timely knowledge translation as the four pillars of effective infodemic management.

In the body of published work on the eHEALS measure, relatively few studies have reported on the relationship between eHEALS scores and specific health behaviors or health outcomes. Neter and colleagues [51] recently reviewed this topic and concluded that although there are some positive associations, additional study on this topic is needed. In the Neter review [51], the most consistent associations seemed to be between eHEALS and health behaviors. For example, Mitsutake and colleagues reported in separate studies the finding of positive association between scores on the general eHEALS measure and health-promoting behaviors (eg, physical activity, healthy eating) and also colorectal cancer screening practice [32,38]. We found that compared to individuals with low CoV-eHEALS scores, those with high CoV-eHEALS scores reported engaging in routine practice of one additional protective behavior (eg, mask wearing, social distancing). These observed relationships are both statistically significant and meaningful from a personal and public health standpoint. Our finding of a positive association between the CoV-eHEALS score and engagement in protective behaviors contributes to our understanding of the eHealth literacy and provides further evidence of the relationship between measures of eHealth literacy (eg, general eHEALS or our CoV-eHEALS) and actual health behaviors and practices.

There are several limitations to consider when interpreting our study findings. First, the results reported here are from a single cross-sectional survey, and thus, we cannot make claims regarding causation. For example, although we did find a negative association between CoV-eHEALS scores and conspiracy beliefs, we cannot be certain whether a higher CoV-eHEALS score led to reduced acceptance of these beliefs or whether a predisposition to conspiracy thinking led to lower CoV-eHEALS scores. Second, it is important to acknowledge that the CoV-eHEALS and COVID-19 KAP measures are based on self-report. The need for further study of the relationship between self-reported eHEALS measures and actual performance has been discussed above. Associations between trajectories of self-reported protective behaviors and COVID-19 cases supports the validity of these self-report measures; however, the precise relationship between self-reported and actual behavior (eg, difference between behavior that is reported to occur “some of the time” vs “almost all of the time”) requires additional study [52]. Third, this survey was conducted during a single, brief time period in one specific country relatively soon after the onset of the COVID-19 pandemic. Further work will be needed to determine how CoV-eHEALS scores may vary across different countries and how this might change over time. Fourth, it is important to note that this survey was performed using a web-based survey format. Although the rates of internet access in the United States are quite high in general and characteristics of our study sample were similar to that of the general US population, the administration of a web-based survey could certainly bias the sample toward individuals with greater familiarity with technology and the internet. Computer and internet use are well-recognized predictors of eHEALS, which we did not specifically assess in this study [22,23,27,30]. As such, our estimate of 29% of the US adult population having low CoV-eHEALS scores should likely be considered a lower bound for this estimate.

Despite these limitations, there are some potentially important implications related to our study findings. We found that although the overall level of coronavirus-related eHealth literacy in this study was relatively high, there still remains a substantial proportion of the US adult population that has low coronavirus-related eHealth literacy; this population might thus be considered at higher risk of negative COVID-19 KAPs. Recent studies assessing the quality of health information available on the internet about COVID-19 have found inconsistent coverage of key public health recommendations with a majority of websites having moderate-to-low quality scores [53,54]. These authors identified substantial opportunities to improve the clarity of presentation of critical health information and argued that broader implementation and adherence to quality standards for presentation of COVID-19−related information available on the internet could be helpful in terms of improving public health literacy on this topic. It is important to note that some major search engines have taken specific steps to improve search simplicity and delivery of high-quality COVID-19−related health information. For example, Google has a dedicated landing page for COVID-19−related information that is displayed following general searches about coronavirus or COVID-19. This landing page highlights key topics (eg, disease trends; access to testing; and health information on symptoms, prevention, and treatments) with summaries of key facts and direct links leading to high-quality information sources. Finally, it is important to recognize that searching for information on the internet can be a complicated and challenging process [55]. Despite the capabilities of modern search engines, people are more effective in finding accurate information if they possess some basic skills in how to find and use information on the web (eg, knowing to “click through” to view the actual website instead of relying upon websites summaries, checking timeliness and quality of information sources, and cross-checking different information sources) [56]. Deficits in such search skills are common in the general population; nevertheless, prior work shows that through web-based training, people can develop these skills and improve their abilities to find high-quality and accurate information on the internet.

Given the consistent associations between CoV-eHEALS scores and COVID-19 KAPs, there may be some benefit in teaching such search skills in general or specifically in terms of searches for COVID-19−related information. In the future, it could be important to assess and track coronavirus-related eHealth literacy at the individual and population levels. Identifying and addressing low coronavirus-related eHealth literacy could prove helpful in improving COVID-19−related knowledge, attitudes, and practices, thereby reducing future illness and deaths during this pandemic.

## Abbreviations

CoV-eHEALS: Coronavirus-Related eHealth Literacy Scale
eHEALS: eHealth Literacy Scale
KAP: knowledge, attitude, and practice
MANOVA: multivariate analysis of variance

## References

[1] Brørs G, Norman C, Norekvål TM (2020). Accelerated importance of eHealth literacy in the COVID-19 outbreak and beyond. Eur J Cardiovasc Nurs.

[2] Chong Y, Cheng H, Chan H, Chien W, Wong S (2020). COVID-19 pandemic, infodemic and the role of eHealth literacy. Int J Nurs Stud.

[3] Sentell T, Vamos S, Okan O (2020). Interdisciplinary perspectives on health literacy research around the world: more important than ever in a time of COVID-19. Int J Environ Res Public Health.

[4] Eysenbach G (2020). How to fight an infodemic: the four pillars of infodemic management. J Med Internet Res.

[5] Coronavirus disease (COVID-19) pandemic. World Health Organization.

[6] Coronavirus Disease 2019 (COVID-19). Center for Disease Control and Prevention.

[7] Clements JM (2020). Knowledge and behaviors toward COVID-19 among US residents during the early days of the pandemic: cross-sectional online questionnaire. JMIR Public Health Surveill.

[8] McFadden S, Malik A, Aguolu O, Willebrand K, Omer S (2020). Perceptions of the adult US population regarding the novel coronavirus outbreak. PLoS One.

[9] Alobuia W, Dalva-Baird N, Forrester J, Bendavid E, Bhattacharya J, Kebebew E (2020). Racial disparities in knowledge, attitudes and practices related to COVID-19 in the USA. J Public Health (Oxf).

[10] Block R, Berg A, Lennon RP, Miller EL, Nunez-Smith M (2020). African American adherence to COVID-19 public health recommendations. Health Lit Res Pract.

[11] Cinelli M, Quattrociocchi W, Galeazzi A, Valensise CM, Brugnoli E, Schmidt AL, Zola P, Zollo F, Scala A (2020). The COVID-19 social media infodemic. Sci Rep.

[12] Tangcharoensathien V, Calleja N, Nguyen T, Purnat T, D'Agostino M, Garcia-Saiso S, Landry M, Rashidian AY, Hamilton C, AbdAllah A, Ghiga I, Hill A, Hougendobler D, van Andel J, Nunn M, Brooks I, Sacco PL, De Domenico M, Mai P, Gruzd A, Alaphilippe A, Briand S (2020). Framework for managing the COVID-19 infodemic: methods and results of an online, crowdsourced WHO technical consultation. J Med Internet Res.

[13] Frenkel S, Decker B, Alba D How the ‘Plandemic’ Movie and Its Falsehoods Spread Widely Online. The New York Times.

[14] Freeman D, Waite F, Rosebrock L, Petit A, Causier C, East A, Jenner L, Teale A-L, Carr L, Mulhall S, Bold E, Lambe S (2020). Coronavirus conspiracy beliefs, mistrust, and compliance with government guidelines in England. Psychol Med.

[15] Rice R (2006). Influences, usage, and outcomes of Internet health information searching: multivariate results from the Pew surveys. Int J Med Inform.

[16] Powell J, Inglis N, Ronnie J, Large S (2011). The characteristics and motivations of online health information seekers: cross-sectional survey and qualitative interview study. J Med Internet Res.

[17] Bangerter L, Griffin J, Harden K, Rutten L (2019). Health information-seeking behaviors of family caregivers: analysis of the Health Information National Trends Survey. JMIR Aging.

[18] Kontos E, Blake K, Chou W, Prestin A (2014). Predictors of eHealth usage: insights on the digital divide from the Health Information National Trends Survey 2012. J Med Internet Res.

[19] Sherman L, Patterson M, Tomar A, Wigfall L (2020). Use of digital health information for health information seeking among men living with chronic disease: data from the Health Information National Trends Survey. Am J Mens Health.

[20] Norman C, Skinner H (2006). eHEALS: The eHealth Literacy Scale. J Med Internet Res.

[21] Paige S, Krieger J, Stellefson M, Alber J (2017). eHealth literacy in chronic disease patients: An item response theory analysis of the eHealth literacy scale (eHEALS). Patient Educ Couns.

[22] Richtering S, Hyun K, Neubeck L, Coorey G, Chalmers J, Usherwood T, Peiris D, Chow CK, Redfern J (2017). eHealth literacy: predictors in a population with moderate-to-high cardiovascular risk. JMIR Hum Factors.

[23] Chung S, Nahm E (2015). Testing reliability and validity of the eHealth Literacy Scale (eHEALS) for older adults recruited online. Comput Inform Nurs.

[24] Milne RA, Puts MTE, Papadakos J, Le LW, Milne VC, Hope AJ, Catton P, Giuliani ME (2015). Predictors of high eHealth literacy in primary lung cancer survivors. J Cancer Educ.

[25] Nguyen J, Moorhouse M, Curbow B, Christie J, Walsh-Childers K, Islam S (2016). Construct validity of the eHealth Literacy Scale (eHEALS) among two adult populations: a Rasch analysis. JMIR Public Health Surveill.

[26] Tennant B, Stellefson M, Dodd V, Chaney B, Chaney D, Paige S, Alber J (2015). eHealth literacy and Web 2.0 health information seeking behaviors among baby boomers and older adults. J Med Internet Res.

[27] Choi NG, Dinitto DM (2013). The digital divide among low-income homebound older adults: internet use patterns, eHealth literacy, and attitudes toward computer/Internet use. J Med Internet Res.

[28] van der Vaart R, van Deursen AJ, Drossaert CH, Taal E, van Dijk JA, van de Laar MA (2011). Does the eHealth Literacy Scale (eHEALS) measure what it intends to measure? Validation of a Dutch version of the eHEALS in two adult populations. J Med Internet Res.

[29] Neter E, Brainin E (2012). eHealth literacy: extending the digital divide to the realm of health information. J Med Internet Res.

[30] Chesser A, Burke A, Reyes J, Rohrberg T (2016). Navigating the digital divide: A systematic review of eHealth literacy in underserved populations in the United States. Inform Health Soc Care.

[31] Stellefson ML, Shuster JJ, Chaney BH, Paige SR, Alber JM, Chaney JD, Sriram PS (2018). Web-based health information seeking and eHealth literacy among patients living with chronic obstructive pulmonary disease (COPD). Health Commun.

[32] Mitsutake S, Shibata A, Ishii K, Oka K (2012). Association of eHealth literacy with colorectal cancer knowledge and screening practice among internet users in Japan. J Med Internet Res.

[33] Li S, Feng B, Liao W, Pan W (2020). Internet use, risk awareness, and demographic characteristics associated with engagement in preventive behaviors and testing: cross-sectional survey on COVID-19 in the United States. J Med Internet Res.

[34] van Prooijen Jan-Willem, van Vugt Mark (2018). Conspiracy theories: evolved functions and psychological mechanisms. Perspect Psychol Sci.

[35] Centers for Disease Control and Prevention - COVID-19.

[36] Toussaint L, Cheadle A, Fox J, Williams D (2020). Clean and contain: initial development of a measure of infection prevention behaviors during the COVID-19 pandemic. Ann Behav Med.

[37] Dynata - The Data Platform for [richer] insights.

[38] Mitsutake S, Shibata A, Ishii K, Oka K (2016). Associations of eHealth literacy with health behavior among adult internet users. J Med Internet Res.

[39] Robinson C, Graham J (2010). Perceived Internet health literacy of HIV-positive people through the provision of a computer and Internet health education intervention. Health Info Libr J.

[40] Do B, Tran T, Phan D, Nguyen HC, Nguyen TTP, Nguyen HC, Ha TH, Dao HK, Trinh MV, Do TV, Nguyen HQ, Vo TT, Nguyen NPT, Tran CQ, Tran KV, Duong TT, Pham HX, Nguyen LV, Nguyen KT, Chang PWS, Duong TV (2020). Health literacy, eHealth literacy, adherence to infection prevention and control procedures, lifestyle changes, and suspected COVID-19 symptoms among health care workers during lockdown: online survey. J Med Internet Res.

[41] Li X, Liu Q (2020). Social media use, eHealth literacy, disease knowledge, and preventive behaviors in the COVID-19 pandemic: cross-sectional study on Chinese netizens. J Med Internet Res.

[42] Bandura A, Pervin L, John O (1999). A social cognitive theory of personality. Handbook of personality (2nd ed.).

[43] Cane J, O'Connor D, Michie S (2012). Validation of the theoretical domains framework for use in behaviour change and implementation research. Implement Sci.

[44] Moores T, Chang Jc (2009). Self-efficacy, overconfidence, and the negative effect on subsequent performance: A field study. Information & Management.

[45] Weinberg B (2009). A model of overconfidence. Pacific Economic Review.

[46] Neter E, Brainin E (2017). Perceived and performed eHealth literacy: survey and simulated performance test. JMIR Hum Factors.

[47] Earnshaw V, Eaton L, Kalichman S, Brousseau N, Hill E, Fox A (2020). COVID-19 conspiracy beliefs, health behaviors, and policy support. Transl Behav Med.

[48] Romer D, Jamieson K (2020). Conspiracy theories as barriers to controlling the spread of COVID-19 in the U.S. Soc Sci Med.

[49] Duplaga M (2020). The determinants of conspiracy beliefs related to the COVID-19 pandemic in a nationally representative sample of internet users. Int J Environ Res Public Health.

[50] Biddlestone M, Green R, Douglas K (2020). Cultural orientation, power, belief in conspiracy theories, and intentions to reduce the spread of COVID-19. Br J Soc Psychol.

[51] Neter E, Brainin E (2019). Association between health literacy, eHealth literacy, and health outcomes among patients with long-term conditions. European Psychologist.

[52] Lazer L, Santillana M, Perlis R, Quintana A, Ognyanova K, Green J, Baum MA, Simonson M, Uslu AA, Chwe H, Druckman J, Lin J, Gitomer A (2020). A 50-State COVID-19 Survey: Report #26: Trajectory of COVID-19-Related Behaviors. The COVID States Project.

[53] Hernández-García I, Giménez-Júlvez T (2020). Assessment of health information about COVID-19 prevention on the internet: infodemiological study. JMIR Public Health Surveill.

[54] Jayasinghe R, Ranasinghe S, Jayarajah U, Seneviratne S (2020). Quality of online information for the general public on COVID-19. Patient Educ Couns.

[55] Russell D (2019). The Joy of Search - A Google Insider's Guide to Going Beyond the Basics.

[56] Russell D, Callegaro M (2019). How to Be a Better Web Searcher: Secrets from Google Scientists. Scientific American.

